# Eating Disorders in Cystic Fibrosis

**DOI:** 10.3390/nu18091374

**Published:** 2026-04-27

**Authors:** Sabina Sabharwal

**Affiliations:** Boston Children’s Hospital, Harvard Medical School, Boston, MA 02115, USA; sabina.sabharwal@childrens.harvard.edu

**Keywords:** cystic fibrosis, eating disorders, body image perception

## Abstract

Background/Objectives: Patients with cystic fibrosis (CF) have been reported to have eating disorders. This can contribute to poor weight gain, which can result in increased morbidity and mortality. The aim of this review is to understand the prevalence and impact of eating disorders (ED) and body image disturbance in the CF population. Methods: A review of the current literature on eating disorders in CF. Results: Disordered eating behaviors appear common in CF. However, it is unclear if the prevalence is greater or similar to that of the general population. Conclusions: Routine screening for eating disorders is important in the care of CF patients to help prolong survival and quality of life in this patient population.

## 1. Introduction

Cystic fibrosis (CF) is an autosomal recessive disease that affects multiple organ systems, most notably the lungs and gastrointestinal (GI) tract. Life expectancy is shortened compared to the non-CF population [1,2]. The majority of people with CF have exocrine pancreatic insufficiency (EPI) and require pancreatic enzyme replacement therapy (PERT) with food intake. Insulin plays a large role in metabolism and eating behavior and is altered in PWCF in CF-related diabetes [3]. Maintaining optimal pulmonary function is directly correlated with a BMI over the 50th percentile [2,4]. Thus, weight is a consistent focus in the care of patients with CF. Starting in infancy, parents and then the patients themselves, as they go through various stages of development into adulthood, are questioned about their diets, feeding/eating habits, and growth curves. Pressure from the medical team and family on weight may further contribute to the entity of disordered eating [5,6].

According to the Diagnostic and Statistical Manual of Mental Disorders (DSM V), anorexia nervosa (AN) is characterized by an intense fear of gaining weight and severe restriction of food intake [7]. Bulimia nervosa (BN) is characterized by recurrent episodes of binge eating, also with compensatory behaviors to prevent weight gain, such as vomiting, abuse of laxatives, or excessive exercise [5,7]. Binge eating disorder is characterized by recurrent episodes of binge eating (at least once a week for three months), without regular compensatory behaviors to prevent weight gain [5,7]. All of the above disorders result from an underlying disturbance in self-perceived body image. Avoidant/restrictive food intake disorder (ARFID) is defined as a lack of interest, avoidance, or concern about eating food with failure to meet nutritional and/or energy needs associated with weight loss, nutritional deficiency, dependence on enteral feeding/oral nutritional supplements, or interference with psychosocial function. Still, unlike AN and BN, there is no altered perception of body image. An increase in social media usage is associated with increased rates of disordered eating in adolescent girls [8]. This societal pressure can also lead to body image issues in the CF population.

The aim of this review is to understand the prevalence and impact of eating disorders and body image disturbance in the CF patient population. This review will focus on older children, adolescents, and adults pwCF.

## 2. Materials and Methods

An extensive review of the literature was conducted through PubMed to search for the most relevant available data on eating disorders in patients with CF, including observational studies, prospective studies, and other reviews, published in the English language at any time of publication. Search terms used for this manuscript were: “cystic fibrosis”, “eating disorders”.

## 3. Results

### 3.1. Existing Research on EDs and Patients with CF

CF is considered a diet-related chronic health condition. There exists an increased risk of EDs in patients with diet-treated chronic illnesses, such as diabetes, celiac disease, and CF [9,10]. In a 2013 meta-analysis, both children and adolescents with CF were found to have increased risk of body image concerns compared to their healthy peers and those with other illnesses such as diabetes [11]. Both patients with CF and their providers have identified body image as an important issue [12]. However, these issues are often not brought up at clinical visits. Several studies have shown that adolescents and young adults (AYAs) with CF have distorted body image and disordered eating [9,13,14].

The prevalence of eating disorders in CF is largely unknown. Some studies have cited a higher prevalence than the general population [15,16]. For example, Pearson et al. reported an incidence of 16.4% of eating disorders in 61 adolescents with CF. Others have reported that the prevalence rates of eating disorders do not differ, or may even be lower than the prevalence rates in the general population [17,18]. For example, Raymond et al. found eating disorders in two of their control groups (N = 43), but none were found in the CF sample (N = 58) [16].

**Figure 1:**
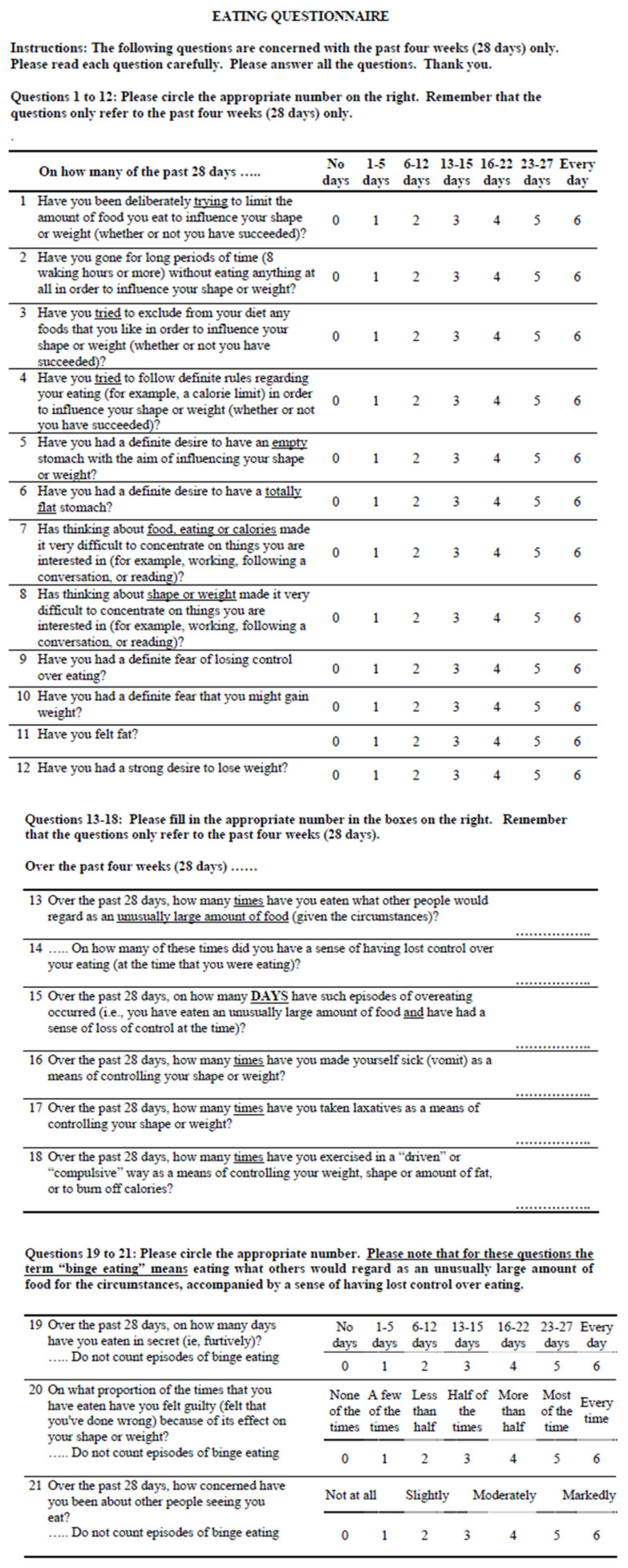

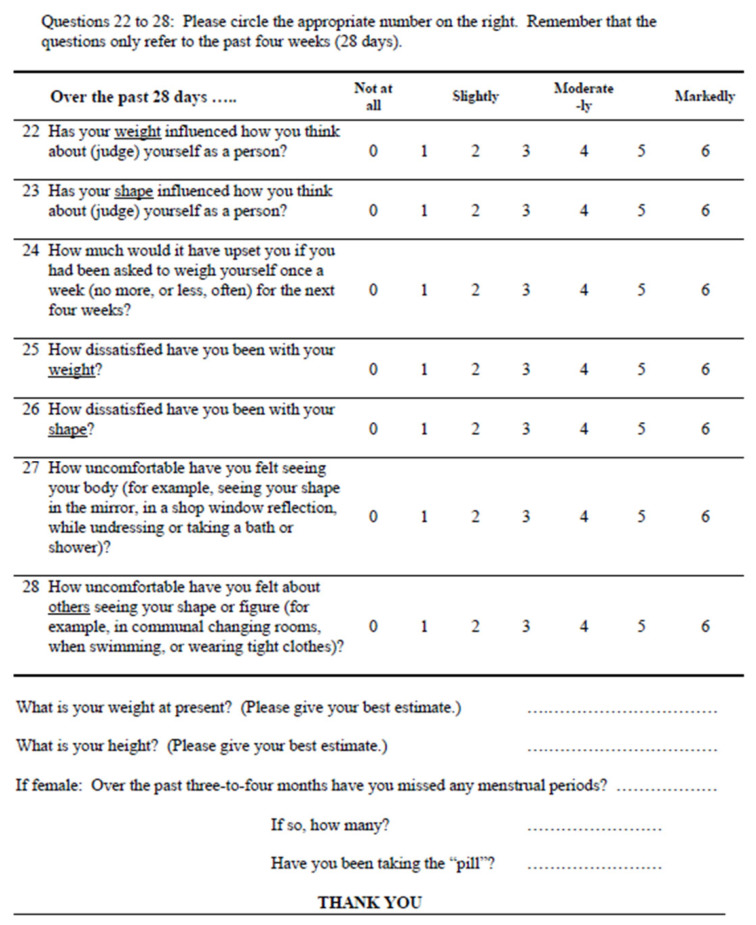
The Eating Disorder Examination Questionnaire (EDE-Q) consists of 28 questions on a 7-point Likert scale (0–6) for adults, measuring frequency or severity of specific behaviors and attitudes over the past 28 days [20,21]. Response options vary by question. It includes four primary subscales: restraint, eating concern, shape concern, and weight concern. The average of the four subscales above generates the global average score. Higher scores indicate greater eating disorder symptom severity, with a score greater than or equal to 4 indicating significant clinical symptoms.

**Figure 2:**
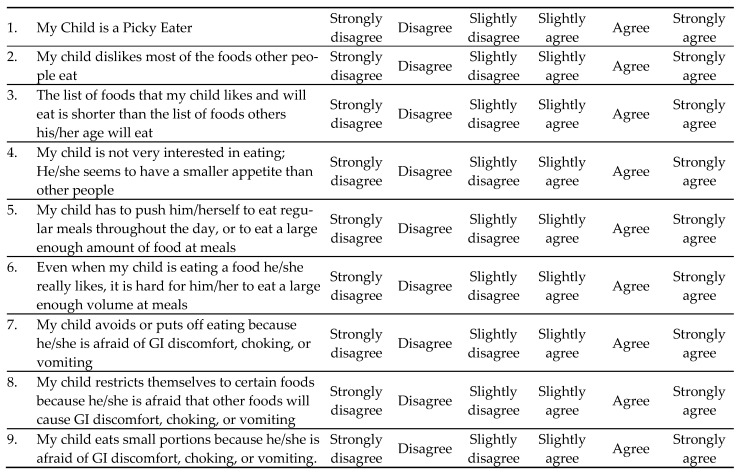
Nine-Item ARFID Screen. Each of the nine items is rated on a 6-point Likert scale: from 0 (“Strongly Disagree”) to 6 (“Strongly Agree”). The total score is calculated by summing all item response scores [22]. The total score ranges from 0 to 45. There are no official cut-off scores; higher scores indicate a greater likelihood and severity of ARFID symptoms.

In 2022, Kass et al. recruited CF patients aged 14–35 years to complete three validated surveys: (1) Eating Disorder Examination Questionnaire (EDE-Q), (2) Nine-Item Avoidant/Restrictive Food Intake Disorder Scale (NIAS), and (3) Cystic Fibrosis Questionnaire-Revised (CFQ-R) [18] (Figure 1 and Figure 2). A clinically significant number of participants screened positive for eating disorders on the EDE-Q and NIAS. It was concluded that more studies will be needed to understand how best to employ these tools in clinical practice for the CF patient population.

In 2023, in another study by Kass et al., a web-based survey was distributed to United States (U.S.)-based CF healthcare providers via CF Foundation list servers, looking at providers’ understanding of issues surrounding disordered eating and body image disturbance in adolescent and young adults with CF (AYAwCF) as well as current screening practices [19]. While most participants felt that screening for both body image disturbance and disordered eating should be standardized in CF care, fewer than one-third of those surveyed felt comfortable screening, and only one-quarter actually screened for various eating disordered behaviors in their practice. Participants reported that provider assessment tools (86%), standardized partnerships with eating disorder specialists (80%), and cystic fibrosis foundation (CFF) or national guidelines (79%) would be helpful to improve screening and counseling.

### 3.2. Considerations for the Multidisciplinary CF Care Team

Historically, AYAwCF maintain a low body weight more easily than those without CF; however, with the added focus on BMI and nutrition as part of their frequent CF multidisciplinary visits, an overemphasis on body image can result. A critical review of studies examining body image among AYAwCF showed that females generally had a more positive body image than males due to their low BMI, while males were more unhappy with their perceived weight and, instead, desired to gain weight [23]. This, in turn, can result in nonadherence with nutrition recommendations and, for example, with adherence to pancreatic enzyme replacement therapy, both of which promote weight gain [11]. No research to date has investigated if or how body image concerns are present in gender-diverse AYAwCF, or among those who represent cultures that are non-Eurocentric- and/or primarily Caucasian [24].

Changing the way multidisciplinary CF teams address weight and BMI concerns may be challenging due to this long-standing emphasis, as previously mentioned. BMI, as a measure, has a number of limitations, including its inability to differentiate fat mass from lean body mass (LBM), with the latter thought to confer a health advantage [23]. Body composition can be measured using several different techniques, including Dual-Energy X-Ray Absorptiometry and Bioimpedance, but has not been studied in the CF population. These measures may become more important as BMI is less emphasized with the use of highly effective modulator therapy (HEMT), which generally results in easier weight gain in patients with CF.

As a multidisciplinary CF team, several aspects of health for patients are reviewed. The medical complications of eating disorders are significant and include multiple body systems [5,25]. Further, these complications can exacerbate baseline CF symptoms. From a pulmonary standpoint, there exists an increased risk of pulmonary muscle wasting, respiratory failure, and spontaneous pneumothorax [25]. From a gastrointestinal standpoint, gastroparesis and constipation can occur [5]. Liver complications occur in undernourished AN patients and can present as hypertransaminasemia [25]. The endocrine system can be affected by pubertal delay and osteoporosis [25]. From a cardiovascular standpoint, arrhythmias, hypotension, and pericardial effusions have been described. Hematologic abnormalities, including cytopenias and anemia, can occur [25]. From a renal standpoint, electrolyte imbalances and refeeding syndrome must be monitored [25]. Certainly, the overlap between several of these symptoms and those commonly seen in CF, including pulmonary dysfunction, osteoporosis, and hypertransaminasemia, can be further exacerbated with the presence of EDs. Further immunodeficiency can further increase the risk for pulmonary infections.

Gastrointestinal complications of EDs can be challenging because they can further impede weight restoration—this includes early satiety with eating, bloating, and a feeling of fullness in constipation [18]. It has been estimated that 41–52% of patients with eating disorders have symptoms suggesting irritable bowel syndrome (IBS) [26,27]. In 2023, the GALAXY study, a multicenter observational study, showed that, using electronic patient-reported outcome measures, over 20% of CF patients report moderate to severe GI symptoms, most commonly constipation and bloating [28]. Given the overlap in symptoms with patients with eating disorders, it would be helpful to identify a possible etiology of these symptoms in order to improve health outcomes, including quality of life.

Micronutrient deficiencies are well described in patients with EDs as well as in patients with CF [25,29]. Depending on the study, 23–58% of eating disorder patients had a vitamin D deficiency [30,31]. In addition, low vitamin D3 may result in a lack of inflammatory response and depressive symptoms in patients with long-term eating disorders [27]. Fat-soluble vitamins A, D, E, and K in CF are commonly attributable to fat malabsorption, related to exocrine pancreatic insufficiency. Several studies have demonstrated the impact of vitamin D insufficiency status on CF pulmonary outcomes [32,33]. Zinc, commonly deficient in ED patients, has been shown to impact appetite and limit progression of cachexia [34]. Zinc has antioxidant and anti-inflammatory effects, and zinc deficiency can be seen in diarrhea and steatorrhea in CF [35]. In a cross-sectional study of 30 infants with CF, diagnosed by newborn screening, plasma zinc was significantly lower in one-third of the infants who improved after initiating PERT [29,35,36].

### 3.3. A New Age and a New Nutritional Model

CF transmembrane conductance regulator (CFTR) modulators are available for 90% of pwCF. HEMT aims to correct the genetic defect in CF. In clinical trials of Trikafta (elexacaftor/tezacaftor/ivacaftor), patients experienced a significant increase in body weight as well as BMI, even resulting in overweight/obesity [18,19,23,24,37,38]. Further, HEMT has been shown to correct EPI [39]. This weight gain may have the unintended consequence of disturbed body image, which could lead to disordered eating behavior in a population with previous difficulty with gaining weight [40,41]. This makes screening and addressing eating behaviors even more imperative.

With the use of HEMT, and weight gain being on the rise in patients with CF, and its negative consequences, including cardiovascular disease and dyslipidemia in adult pwCF, it is important to reframe our discussion around BMI to a weight-neutral approach [37,38,39,40,41,42]. In treating overweight and obesity, a weight-neutral approach (WNA) favors improving overall health outcomes versus losing weight. Examples of outcomes in this approach include improved physical activity, diet quality, and cholesterol levels, which in turn can result in a lower weight and BMI [24]. While no studies of WNA in patients with CF exist, these approaches are becoming popular in patients who struggle with a distorted body image and disordered eating [24,41,42,43].

## 4. Discussion

There has been improved survival and quality of life in patients with CF. The historic emphasis on BMI and current improvement in the ability to gain weight with HEMT place these patients at an increased risk for body image concerns, disordered eating, and diagnostic eating disorders [24]. Utilizing screening tools as CF providers is important for assessing body image disturbances and eating disorders. However, many of the tools used to assess for eating disorders have not been validated in the CF population, particularly since the advent of HEMT [19]. In their best practice guidelines, the European CF Society recommends a personalized and multidisciplinary approach to care, including attention to body image disturbance and disordered eating, as it may impact the overall treatment and prognosis of patients with CF. Validated screening tools and a multidisciplinary WNA may further improve quality of life in the CF population [41,44].

The WNA prioritizes emotional and physical wellness versus weight loss while also aiming to destigmatize and end discrimination for people with larger bodies [24,45]. The health at every size (HAES) approach has been advocated for by Lyons et al. regarding eating and body image in CF [24,46]. This is proposed to involve several members of the CF care team and empowers weight-inclusive language—given that weight gain is now associated with HEMT—and nutritional emphasis on mindful eating. There is little research occurring regarding looking at the implementation of WNAs in chronic illnesses managed by diet; no studies looking at this include PwCF. In order for multidisciplinary CF care teams to implement these WNAs, continuing education around this is necessary [24]. The Association for Size Diversity and Health (ASDAH) offers HAES training. Similarly, for eating disorders, educational resources include the National Eating Disorder Association (NEDA), the International Association of Eating Disorders Professionals (IAEDP), the Eating Recovery Center (ERC), and The Emily Program [24].

Intervening early is key to preventing disordered eating behaviors, which often appear in adolescence. Caregiver mental health is a key driver of improved mental and physical health in the child, and it is important to address parental stress around meals [24]. It is important for parents to feel confident in empowering their children to make healthy decisions around eating and mealtimes. Brief questionnaires can assess eating behaviors during routine CF clinic visits. The Behavioral Pediatrics Feeding Assessment Scale (BPFAS) is a parent-reported questionnaire that looks at child-feeding practices and their approach to strategies around mealtime, with scores being immediately available at the time of the visit so that recommendations can be made [47,48]. Another example of an assessment tool is the NIAS that can be used to assess for ARFID [22].

In a recent review on eating disorders in CF, it was noted that the most common psychiatric comorbidities associated with CF—anxiety and depression—are also prevalent in EDs [49,50]. Dysthymia, a mild, long-lasting form of depression, has been described in pwCF and atypical eating disorders [51].

The CF Eating Attitudes and Behaviors Scale (CFEAB) was developed by Randelsome et al. [52]. It is a 24-item self-report scale that measures three factors of disordered eating: appetite—expressing general pleasure with eating behaviors; disturbed eating and behaviors, related to eating disordered psychopathology; and a desire for thinness and weight loss, such as feelings about appearance and weight management [53]. A cross-sectional study published in 2023 validated the CFEAB in an adult CF population of 132 patients [50].

It remains very important to acknowledge the key characteristics of CF as a disease state that further characterize disordered eating in this population. Ports, enteral tubes, and supplemental oxygen may affect body image [3,5]. Pancreatic-sufficient patients tend to express more body image and body weight concerns than pancreatic-insufficient patients [54]. In a study examining female pwCF and CF-related diabetes, participants expressed dissatisfaction with their body appearance and self-esteem [55]. Case reports suggest that female pwCF and AN have a diminished FEV1 [55,56]. Often overlooked symptoms of body weight manipulation through misuse of pancreatic enzyme replacement therapy and insulin have also been reported in pwCF [5,51].

The medical complications of eating disorders are reviewed above, but it is important to understand that malnutrition can exacerbate mental health disorders. Anxiety and depression are common psychiatric comorbidities in eating disorders [57]. Studies involving individuals with CF have found a prevalence of depression of 8–29% in children and adolescents and 13–33% amongst adults, while anxiety in adults ranges from 30 to 33% [58,59]. Furthermore, while anxiety and depression may contribute to the development of an eating disorder, the eating disorder, in turn, may aggravate underlying anxiety and depression [60,61].

Finally, in 2024, the European Society of Clinical Nutrition and Metabolism (ESPEN), the European Society for Pediatric Gastroenterology Hepatology and Nutrition (ESPGHAN), and the European Cystic Fibrosis Society (ECFS) together put forth guidelines on CF nutrition care for infants, children, and adults, which reflected a change in the landscape of CF nutrition [62]. It is important to note that maintaining a healthy weight can avert chronic conditions, necessitating the inclusion of fruits, vegetables, and wholegrains in one’s diet while limiting added sugar, salt, and saturated fat. This removal of the legacy CF diet and a weight-neutral approach in the age of HEMT could lead to less emphasis being placed on eating and weight and, as such, lead perhaps to a decline in the prevalence of disordered eating.

## 5. Conclusions

In this review, current information on eating behaviors and disordered eating in PWCF was reviewed, and important future areas were examined. Although disordered eating behaviors, including eating disorders, are common in pwCF, it is unclear if this prevalence is higher than that of the general population. There exist limited high-quality studies looking at eating disorders in pwCF, and small studies make generalizability difficult [3]. Although several factors identified above might contribute to a higher incidence of eating disorders in the CF population, causality remains unproven. Both heterogeneity of screening and the assessment measures used also make it difficult to form a direct comparison across studies [45]. Because of the important role that body weight has on pulmonary function, it is important that any factors affecting this be addressed and understood. With the advent of HEMT and weight gain in pwCF, a shift to a WNA approach may be favored by multidisciplinary CF care teams. Consistent messaging and support from this team is key [62,63,64]. Still, more research in terms of larger studies using CF-specific tools is required to better understand how to best screen for and identify these disordered eating behaviors earlier. Referrals to colleagues in adolescent medicine and psychology may be helpful so as to expand the multidisciplinary CF team. Currently, there are no reliable, validated screening tools in the United States that identify disordered eating in pwCF, thus leaving a gap in the current CF care model in terms of improving quality of life for pwCF.

## Data Availability

No new data were created or analyzed in this study.
